# Digital health technologies in pain management for patients with endometriosis: A scoping review

**DOI:** 10.1371/journal.pone.0345756

**Published:** 2026-03-25

**Authors:** Xinrui Wang, Hongyan Wang

**Affiliations:** 1 School of Nursing, Zhejiang Chinese Medical University, Hangzhou, Zhejiang Province, China; 2 Gynecology Department, Women’s Hospital School of Medicine Zhejiang University, Hangzhou, Zhejiang Province, China; Athens Medical Group, Psychiko Clinic, GREECE

## Abstract

**Objective:**

This scoping review aimed to map the evidence on how digital health technologies are used for pain management in endometriosis.

**Methods:**

This scoping review adhered to the Joanna Briggs Institute methodological framework and followed the preferred reporting items for systematic reviews and meta-Analyses extension for scoping reviews (PRISMA-ScR) guidelines. PubMed, Web of Science, Embase, CINAHL, Cochrane Library, China National Knowledge Infrastructure (CNKI), Wanfang Database, and China Biomedical Literature Database were searched. The search timeframe was from the establishment of each of the databases to June 6, 2025. Studies focusing on patients aged 18 years and older with endometriosis, and examining the application and potential impact of digital health technologies in pain management, were included in the study. Collated in data-extraction tables were authorship information, publication date, country, study design, study population, sample size, application forms, content elements, intervention duration, and outcome indicators.

**Results:**

A total of 18 papers were included. Remote network platforms, mobile applications, virtual reality, and artificial intelligence are some of the application forms of digital health technologies. The content elements covered pain diagnosis, pain assessment and monitoring, pain intervention, and pain education, while the outcome indicators mainly included pain-related indicators, psychosocial indicators, and feasibility evaluation.

**Conclusion:**

This scoping review systematically synthesizes the forms, core contents, and clinical outcomes of digital health technologies for managing endometriosis-related pain, suggesting their preliminary feasibility and potential benefits. Building on these findings, future research should focus on three priorities: refining objective assessment metrics, using these technologies to build more scientifically comprehensive personalized pain management plans, and implementing long-term studies to enhance intervention outcome optimization.

## Introduction

Endometriosis is a benign estrogen-dependent inflammatory disease characterized by the presence, growth, and infiltration of endometrial tissue outside the uterus [1]. It is estimated that approximately 2%−10% of women of reproductive age worldwide are affected by endometriosis [2]. These symptoms, which primarily include dysmenorrhea, chronic pelvic pain, and dyspareunia, are collectively designated as endometriosis-related pain and have a marked adverse effect on patients’ quality of life [3,4].

Currently, no effective treatment exists for endometriosis-associated pain (EAP). Guidelines recommend long-term management with medication, surgery, and psychotherapy [5]. However, the complexity of pain poses major challenges: assessments lack precision and real-time monitoring, treatment options remain limited, outcomes are often unsatisfactory with high relapse after discontinuation, and patients frequently show low self-management engagement and poor access to care [6–8].

Digital health technologies refer to digital technologies that enable the provision of remote health-related services to patients, encompassing electronic health records, telemedicine, artificial intelligence, and various device-supported mobile health technologies [9]. For the purpose of this review, we have adopted a more focused operational definition, specifically including interactive tools that directly engage patients in their care, while excluding tools used exclusively by healthcare providers. Digital health technologies encompass the remote communication function between patients and providers inherent in telemedicine, and extend beyond it by aiming to empower patients through features that support continuous engagement and self-management. Within the context of chronic pain management, these technologies deliver comprehensive support, encompassing remote patient monitoring, clinical decision support, personalized therapeutic interventions, and peer-based social engagement [10,11]. These characteristics provide a novel strategy for EAP management: patients with endometriosis are predominantly young women, who show high acceptance of digital health technologies in diverse forms; the real-time monitoring function of these technologies can accurately match the chronically fluctuating symptomatic nature of EAP; at the same time, they can break through barriers to healthcare such as geographical limitations, thereby offering more continuous, flexible, and personalized support for the long-term management of EAP. Despite their growing use in chronic pain management, the application of these technologies to EAP remains limited and largely exploratory. Significant variability exists in aspects such as its application forms, specific content, assessment tools, and effectiveness outcomes, with a lack of systematic research and synthesis. This study synthesizes and analyzes existing research on the use of digital health technologies in pain management for patients with endometriosis, aiming to provide insights for further research in this field and the optimization of clinical practice.

## Methods

We reported this review according to the preferred reporting items for systematic reviews and meta-analyses extension for scoping reviews (PRISMA-ScR) [12]. The protocol for this review was registered in OSF (Open Science Framework- https://osf.io/a3wdb).

### Ethical considerations

This study is a secondary analysis of previously published literature and does not involve the direct collection of any new raw data from human participants or animals. Therefore, this study does not require separate approval from an institutional ethics review committee.

### Research question

Research questions: (i) what are the application forms of digital health technologies of endometriosis patients? (ii) what are the content elements of digital health technologies of endometriosis patients? (iii) what are the outcome indicators for digital health technologies in pain management of endometriosis patients? (iv) what are the intervention effects of digital health technologies of endometriosis patients?

### Information source and search strategy

A search was conducted in the electronic databases PubMed, CINAHL, Web of Science, Embase, Cochrane Library, China Knowledge Network, Wanfang Database, and China Biomedical Literature Database, covering literature in both English and Chinese published up to June 6, 2025. References were also tracked throughout the review process. To ensure search precision, the strategy was constructed by combining medical subject terms and keywords, and the English database was searched with PubMed as an example of search formula:

1 (“Digital Health”[MeSH Terms] OR “digital health technolog*”[Title/Abstract] OR ‘‘digital therapeutics’’[Title/Abstract] OR “telemedicine”[MeSH Terms] OR “Mobile Health”[Title/Abstract] OR “mhealth”[Title/Abstract] OR “telehealth”[Title/Abstract] OR “ehealth”[Title/Abstract] OR “telecare”[Title/Abstract] OR “Internet-Based Intervention”[MeSH Terms] OR “Web-based Intervention”[Title/Abstract] OR “Online Intervention”[Title/Abstract] OR “website”[Title/Abstract] OR “internet”[Title/Abstract] OR “Mobile Applications”[MeSH Terms] OR “Portable Software App”[Title/Abstract] OR “mobile phone’’[Title/Abstract] OR “Wearable Electronic Devices”[MeSH Terms] OR “electronic handheld device”[Title/Abstract] OR “Virtual Reality”[MeSH Terms] OR “virtual”[Title/Abstract] OR “internet of things”[MeSH Terms] OR “Artificial Intelligence”[MeSH Terms] OR “AI”[Title/Abstract])
2 (“endometriosis”[MeSH Terms] OR “endometriosis”[Title/Abstract] OR “endometrioma”[Title/Abstract] OR “endometriomas”[Title/Abstract])
3 (“pain”[MeSH Terms] OR “pain*”[Title/Abstract] OR “dysuria”[MeSH Terms] OR “dysuria”[Title/Abstract] OR “dyspareunia”[MeSH Terms] OR “dyspareunia”[Title/Abstract] OR “dyschezia”[Title/Abstract] OR “abdominal pain”[Title/Abstract] OR “chronic pelvic pain”[Title/Abstract])
4 #1 AND #2 AND #3

Chinese databases were searched on China National Knowledge Infrastructure, for example, with the search formula: (Subject: “endometriosis”) and (Subject: “pain” or “dysmenorrhea” or “chronic pelvic pain” or “dyspareunia” or “acute abdominal pain” or “dyschezia” or “dysuria”) and (Subject: “mobile health” or “telehealth” or “digital health” or “e-health” or “web-based intervention” or “internet” or “computer” or “website” or “mobile platform” or “software” or “online” or “mobile application” or “WeChat” or “wearable device” or “virtual reality” or “artificial intelligence” or “big data” or “internet of things”).

### Inclusion and exclusion criteria

We established the inclusion criteria according to the PCC principle [13]. (i) Participants (P): patients with endometriosis (18 years old and older); (ii) Concept (C): involving the original literature on the application and potential impact of digital health technologies in pain management of endometriosis patients; (iii) Context (C): the various settings where pain management is implemented, such as communities, nursing institutions or hospitals. The type of study was limited to original quantitative, qualitative, and mixed-methods studies. Exclusion criteria: (i) studies unrelated to digital health technologies; (ii) studies unrelated to the pain management of endometriosis; (iii) studies focusing solely on strictly provider-facing tools, website use tracking, general health or fitness trackers; (iv) research protocols, policy opinions, guidelines, etc.; (v) full text not available [14].

### Study selection process

After removing duplicates using EndNote X9 software, the study screening was conducted independently by two researchers in two phases, strictly adhering to the predefined inclusion and exclusion criteria. First, titles and abstracts of all retrieved records were reviewed. Subsequently, the full texts of studies deemed potentially eligible were further assessed for final inclusion. To ensure the consistency and reliability of the screening process, an inter-rater reliability check was performed on a random sample comprising 15% of records from each phase (n = 179 in the title/abstract screening phase; n = 26 in the full-text screening phase). Any disagreements identified during this calibration check or throughout the overall screening process were first resolved through discussion between the two primary reviewers. In cases where consensus could not be reached, a third senior researcher was consulted to make a final decision.

### Data extraction

Following the JBI methodology, we first conducted a pilot test of the data extraction tool on three randomly selected full-text articles to ensure its reliability and consistency. Subsequently, two reviewers independently reviewed full-text and extracted data into Microsoft Excel. All core extraction variables extracted in this review were pre-specified in the OSF including general information such as author, publication date, country, study design, study population, sample size, application forms, content elements, intervention duration, and outcome indicators.

## Results

**Fig 1:**
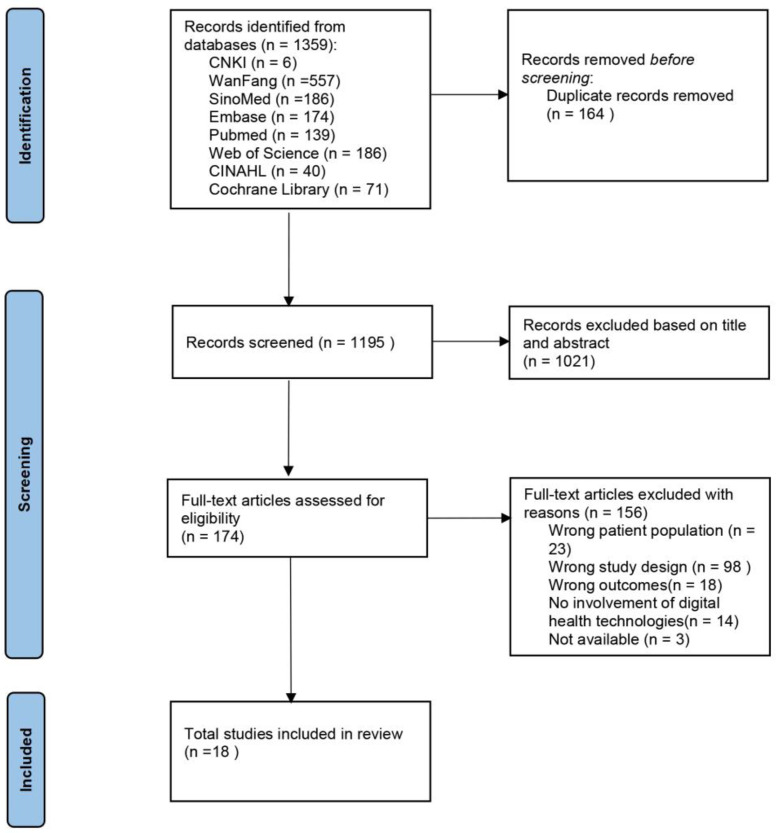
Flow chart of study screening.

Following an initial database search yielding 1359 records, 174 studies were selected after duplicate removal and title/abstract screening. Full-text assessment resulted in the final inclusion of 18 publications (please see Fig 1).

### Study characteristics

Eighteen included studies were published between 2020 and 2025, with the distribution of regional sources as follows: Germany (n = 4) [15–18], Canada (n = 3) [19–21], France (n = 3) [22–24], the United States (n = 2) [25,26], China (n = 2) [27,28], the United Kingdom (n = 2) [29,30], Australia (n = 1) [31] and the Netherlands (n = 1) [32]. The included studies comprised randomized controlled trials (n = 4) [18,23,24,31], quasi-experimental study (n = 3) [15,27,28], qualitative studies (n = 3) [16,21,30], mixed studies (n = 4) [19,20,26,29] and observational studies (n = 4) [17,22,25,32]. The basic characteristics of the included literature are shown in Table 1. Table 2 presents a mapping of digital health technologies for pain management to core functions, categorized by technology type.

**Table 1:** Basic characteristics of the included literature (n = 18).

Author	Year	Country	Study design	Study population	Sample size	Application forms	Duration	Content elements	Outcome indicators
Pakebuschet al. [15]	2025	Germany	Quasi-experimental study	D	23	Virtual reality	14 weeks	③	a、b
Zugaj et al. [16]	2024	Germany	Qualitative study	A	10	Mobile application	4 weeks	③	c
Rohloff et al. [17]	2024	Germany	Observational study	A	64/42	Mobile application	2 weeks	③	b
Rohloff et al. [18]	2024	Germany	RCT	A	61/61	Mobile application	12 weeks	③	a、b
Parmar et al. [19]	2025	Canada	Mixed study	E	32	Remote network platform	Not reported	④	c
Miazga et al. [20]	2024	Canada	Mixed study	D	10	Remote network platform	8 weeks	③	a、b
Howard et al. [21]	2023	Canada	Qualitative study	B	6	Remote network platform	6 weeks	③	c
Breton et al. [22]	2025	France	Observational study	A	92/149	Mobile application	3 months	③	a
Merlot et al. [23]	2023	France	RCT	D	51/51	Virtual reality	6-day follow-up	③	a、b
Merlot et al. [24]	2022	France	RCT	D	23/22	Virtual reality	4-hour follow-up	③	a
Kiser et al. [25]	2024	the United States	Observational study	A	473	AI	Not reported	①	a
Guan et al. [26]	2022	the United States	Mixed study	B	15	Mobile application	7-14 days	②	c
Zhang [27]	2023	China	Quasi-experimental study	C	63/63	Remote network platform	6 months	③	a、b、c
Feng et al. [28]	2020	China	Quasi-experimental study	C	48/48	Remote network platform	5 months	④	b
Abdulai et al. [29]	2022	the United Kingdom	Mixed study	E	12	Remote network platform	Not reported	④	c
Gater et al. [30]	2020	the United Kingdom	Qualitative study	B	31	Mobile application	7 days	②	c
Lutfi et al. [31]	2023	Australia	RCT	D	7/8/4	Remote network platform + Virtual reality	2-day follow-up	③	a
Barneveld et al. [32]	2021	the Netherlands	Observational study	B	5	Mobile application	28 days	②	c

Note: **Study population:** A. Endometriosis B. EAP C. Endometriosis-related dysmenorrhea D. Endometriosis-related pelvic pain E. Endometriosis-related dyspareunia S**ample size:** “1/2” corresponds to the sample sizes of the intervention group/ control group; “1/2/3” corresponds to the sample sizes of the three intervention groups, which are the intervention group 1, intervention group 2, and control group in sequence. **Content elements:** ①Pain diagnosis ②Pain assessment and monitoring ③Pain intervention ④Pain education **Outcome indicators:** a. Pain-related indicators b. Psychosocial indicators c. Feasibility evaluation

**Table 2:** Digital health technologies for pain management mapped to core functions by technology type.

	Pain diagnosis (n = 1)	Pain assessment and monitoring (n = 3)	Pain intervention (n = 11)	Pain education (n = 3)
**Artificial intelligence** **(n = 1)**	[25]			
**Virtual reality** **(n = 4)**			[15,23,24,31]	
**Mobile application** **(n = 7)**		[26,30,32]	[16–18,22]	
**Remote network platform** **(n = 7)**			[20,21,27,31]	[19,28,29]

### Application forms

Currently, the applications of digital health technologies for pain management in patients with endometriosis fall into four main categories:①Remote network platforms [19–21,27–29,31]: Relevant services can be provided by leveraging existing website platforms or developing dedicated health education websites. ②Mobile application [16–18,22,26,30,32]: With mobile devices such as smartphones and tablets as the primary carriers, these apps integrate functions including real-time symptom recording, health education, exercise guidance and psychological support, covering the full-process needs of patients from symptom monitoring to daily health management. ③Virtual Reality (VR) [15,23,24,31]: Highly immersive virtual scenarios are created via VR headsets, delivering multi-sensory stimulation to patients through visual, auditory and other sensory channels. ④Artificial Intelligence (AI) [25]: Machine learning algorithms are applied to analyze pain-related data, so as to assist clinicians in clinical disease diagnosis.

### Content elements

Digital health technologies contribute to pain management in patients with endometriosis across four core dimensions: ①Pain assessment and monitoring. This dimension primarily relies on patient-reported outcome measures. Two studies developed the Endometriosis Symptom Diary (ESD), the Endometriosis Impact Scale (EIS), and the Endometriosis Daily Diary (EDD) to assess the type, location and duration of pain in patients with endometriosis, as well as the impact of pain on daily activities and mood [26,30]. One study applied the *MEASuRE* tool for dynamic monitoring of pain symptoms in patients with EAP. By administering daily symptom questionnaires to patients over a 28-day period, it systematically collected data on pain, sleep, diet, and medication use, thereby enabling accurate capture of pain symptoms and their temporal changes [32]. ②Pain diagnosis. One study conducted cluster analysis on 155 pain locations, identifying 15 distinct pain site clusters. It then leveraged machine learning algorithms to calculate the relative risk of endometriosis based on combinations of pain locations and types, revealing specific diagnostic predictive patterns [25]. ③Pain intervention. Two studies involved psychological interventions incorporating digital storytelling, group meditation, and emotion regulation training [20,21]. Three studies adopted VR-based interventions, using headsets to deliver therapeutic virtual environments featuring natural sounds and simple visual stimuli, which provided diverse relaxation or activity scenarios [15,23,24]. One study designed three study arms: a 1-hour supervised telehealth exercise group (combining cardiopulmonary interval training and lumbopelvic stabilization), a VR group (10 minutes of pain distraction plus 50 minutes of aerobic exercise via VR applications), and a control group without structured exercise. This study compared the immediate pelvic pain relief effects of the two digital health-supported exercise modalities against the control group [31]. Four studies utilized the *Endo* or *School of Endo* programs to deliver multimodal self-management for EAP, including exercise guidance, symptom tracking, and social support [16–18,22]. Another study adopted a blended online-offline traditional Chinese medicine program to guide patients in acupoint massage. [27]. ④Pain education. One study disseminated educational materials on the pathophysiological mechanisms and clinical management of endometriosis-related dysmenorrhea [28]. Additionally, dedicated educational websites focusing on dyspareunia and pelvic pain were shown to further enhance patients’ access to targeted health information [19,29].

### Outcome indicators

The outcome indicators for digital health technology-based interventions pertain to three domains: pain-related indicators, psychosocial indicators and feasibility assessment. ①Pain-related indicators included pain intensity, frequency of pain medication use and impact of pain on function. Eight studies evaluated pain management through effective tools such as the Visual Analogue Scale (VAS), the Numeric Rating Scale (NRS) and the Pain Self-Efficacy Questionnaire (PSEQ). Of these, seven studies reported observed improvements associated with digital health technology-based interventions, including reduced pain levels, lower rates of pain medication utilization, diminished pain-related disruption to daily life, and enhanced self-efficacy in patients [15,18,22–24,27,31]. Notably, one study showed no significant effect on alleviating pain intensity in the short term [20]. ②Psychosocial indicators: quality of life, anxiety, depression, sleep quality, stress and fatigue. Seven studies reported improvements in patient quality of life associated with the use of digital health technologies, as assessed by tools including the Endometriosis Health Profile-5 (EHP-5), Endometriosis Health Profile-30 (EHP-30), World Health Organization Quality of Life-100 (WHOQOL-100), Spitzer Quality of Life Index (SQLI), and Short-Form Health Survey (SF-36) [17,18,20,22,23,27,28]. Evidence from four studies supported the role of digital health interventions in improving negative emotions. Measurements were conducted using standardized tools, namely the Patient Health Questionnaire-9 (PHQ-9) and the Generalized Anxiety Disorder-7 (GAD-7) scale [15,18,22,27]. ③Feasibility assessment: the feasibility of implementing digital health technologies was uniformly confirmed in eight studies through through the analysis of patient satisfaction, adherence and user experience [16,19,21,25,26,29,30,32]. To more clearly illustrate the key findings of the included studies, the detailed information on interventions, types of pain and outcome directions was further tabulated separately and presented in Tables 3–7.

**Table 3:** Summary of findings for digital health technologies in included RCTs.

Author	Digital health intervention information		Types of pain	Effect on pain-related indicators	Effect on psychosocial indicators
	Intervention group	Comparison			
Rohloff et al. [18]	Endo-App (≥5–10 min/ day)	Routine care	Not explicitly stated	Significant reduction in pain disability index and improvement pain self-efficacy	Significant improvement in quality of life, fatigue, and depression; no significant difference in anxiety
Merlot et al. [23]	Endocare (20 min/session, ≤ 2 sessions daily)	Without therapeutic stimulations	Chronic pelvic pain Dysmenorrhea Deep dyspareunia Dysuria Dyschezia	Significantly reduced pain intensity at all time points vs control; Trend for a greater reduction in medication use, but no significant difference between groups	No significant difference between groups
Merlot et al. [24]	Endocare (20 min)	Nature sounds in 2D environment		Significantly reduced pain intensity at all time points vs control	None
Lutfi et al. [31]	Group 1: A self-managed intervention consisting of a 10-minute pain-distraction experience and 50-minute aerobic exercise via VR applications Group 2: A supervised 1-hour exercise intervention delivered via telehealth	Without specific exercise	Chronic pelvic pain	Greater reduction in pain intensity in Groups 1and 2 vs. control, but no significant intergroup difference	None

**Note: Endo-App:** A medical-grade application developed by Endo Health GmbH in Germany, incorporating psychosocial support, exercise therapy, nutritional therapy, and more. **Endocare:** A virtual reality digital therapeutic tool integrating auditory (e.g., alpha/theta binaural beats, nature-based sounds) and visual (e.g., bilateral alternative stimulations) components in a 3D VR environment.

**Table 4:** Summary of findings for digital health technologies in included quasi-experimental studies.

Author	Digital health intervention information		Types of pain	Effect on pain-related indicators	Effect on psychosocial indicators
	Intervention group	Comparison			
Pakebusch et al. [15]	Group 1: VR headset with relaxation applications Group 2: VR headset with activity applications (15–20 min/ session; 3 times weekly)	Routine care	Chronic pelvic pain	Significant improvement in mean pain score, pain catastrophizing, and pain disability index for VR-R (vs. baseline and control) and in pain catastrophizing, and pain disability index for VR-A (vs. baseline only)	Significant improvement in depression, anxiety, and stress for VR-R (vs. baseline and control) and for VR-A (vs. baseline only)
Zhang. [27]	Internet-based traditional Chinese medicine nursing	Routine care	Dysmenorrhea	Significant reduction in dysmenorrhea symptom severity	Significant improvement in quality of life, depression and anxiety
Feng et al. [28]	Continuous nursing intervention based on online interactive platforms	Routine care	Dysmenorrhea	None	Significant improvement in quality of life

**Table 5:** Summary of findings for digital health technologies in included qualitative studies.

Author	Digital health intervention information	Types of pain	Feasibility
Zugaj et al. [16]	Endo-App	Chronic pelvic pain	Mixed outcomes: Positive experiences alongside notable usability barriers
Howard et al. [21]	Digital storytelling through online workshops	Chronic pelvic pain Dysmenorrhea	Positive outcomes in emotional healing and social connectedness
Gater et al. [30]	The Endometriosis Symptom Diary (ESD) and Endometriosis Impact Scale (EIS)	Dysmenorrhea Deep dyspareunia Dysuria	Positive outcomes in content effectiveness and ease of use

**Table 6:** Summary of findings for digital health technologies in included mixed studies.

Author	Digital health intervention information		Types of pain	Effect on pain-related indicators	Effect on psychosocial indicators	Feasibility
	Intervention group	Comparison				
Parmar et al. [19]	An educational website About dyspareunia	None	Deep dyspareunia	None	None	Positive outcomes for website functionality and emotional empowerment
Miazga et al. [20]	Virtual mindfulness-based stress reduction course through zoom platform (8 × 2.5h sessions)	Pre-intervention	Chronic pelvic pain Dysmenorrhea Deep dyspareunia	No signicant change in pain-related indicators during study	Significant improvement in quality of life	None
Guan et al. [26]	An endometriosis symptom diary	None	Chronic pelvic pain Dysmenorrhea Deep dyspareunia	None	None	High ease of implementation
Abdulai et al. [29]	An educational website about dyspareunia	None	Deep dyspareunia	None	None	High user satisfaction with website usability, content clarity, and interface comfort

**Table 7:** Summary of findings for digital health technologies in included observational studies.

Author	Digital health intervention information		Types of pain	Effect on pain-related indicators	Effect on psychosocial indicators	Feasibility
	Intervention group	Comparison				
Rohloff et al. [17]	Endo-App	Without adopting the app	Chronic pelvic pain Dysmenorrhea deep Dyspareunia	Significant reduction in pain scores	Significant improvement in quality of life	None
Breton et al. [22]	School of Endo-APP	Without adopting the app	Chronic pelvic pain Dysmenorrhea Deep dyspareunia Dysuria Dyschezia Neuropathic pain Endo belly	Significant improvement in neuropathic pain and endo belly	Significant improvement in quality of life, depression and anxiety	None
Kiser et al. [25]	Pain-related data clustering analysis based on artificial intelligence	None	Chronic pelvic pain Dysmenorrhea Dyspareunia Dysuria Dyschezia	None	None	Significant for predictive efficacy and key pain feature identification
Barneveld et al. [32]	Random pain assessment through MEASuRE-APP (ten times daily)	None	Chronic pelvic pain	None	None	Strong functional integrity, but with adherence issues

**Note: School of Endo-APP**: an application developed based on cognitive behavioral therapy and the endometriosis health profile, integrating multidisciplinary self-management tools such as disease education and dietary guidance.

## Discussion

### Preliminary success and potential of digital health technologies in endometriosis pain management

The findings of this study indicate that digital health technologies for pain management in patients with endometriosis are available in diverse forms, including remote network platforms, mobile applications, VR, and AI. These technologies offer multiple pathways for pain management, primarily involving assessment and monitoring, diagnosis, intervention, and education. Web-based patient-report outcome measurement tools generate continuous, real-time data, which quantifies and tracks patients’ pain status while minimizing biases associated with long-term recall [30]. The *Endo* app delivers a interactive and engaging pain management platform for patients, which helps alleviate pain and negative emotions, and to some extent, overcomes the temporal and spatial limitations of traditional interventions [18]. Professional pain education websites provide convenient, multifunctional support for patients, facilitating a better understanding and self-management of pain, as well as enhancing patients’ self-efficacy and confidence in seeking medical care [29]. Digital follow-up interventions can maintain patients’ long-term health outcomes without compromising the effectiveness of core therapeutic measures [33]. Collectively, these findings highlight digital health technologies as a valuable complement to traditional pain management strategies: they enhance the flexibility and accessibility of healthcare services, while delivering more continuous and personalized follow-up care and support for patients with chronic pain related to endometriosis.

### Future directions of digital health technologies in endometriosis pain management

#### Incorporating objective outcome measures for multidimensional assessment.

Outcome measures in pain management are multidimensional: In addition to pain intensity and its impact on daily activities and psychological well-being, some studies also incorporate associated symptoms such as fatigue and sleep disturbances. This multidimensional assessment approach not only reflects treatment outcomes more accurately and comprehensively but also enables subsequent interventions to better address patients’ core needs, ultimately driving overall improvements in quality of life. However, these indicators remain largely reliant on patient self-reports and are susceptible to subjective factors including individual emotional states and cognitive biases, which may lead to infrequent reporting or incomplete data entry [32]. A small number of studies have introduced physiological indicators to assist in evaluation [18,20], such as using the frequency of pain-related medication use as an indirect measure of pain control. Yet medication use may be influenced by factors such as healthcare policies and patient preferences, making it difficult to fully reflect the actual pain status. Accumulating evidence indicates that wearable devices, such as smart bands and sensors, are being used to continuously monitor physiological signals including heart rate, heart rate variability, and physical activity, thereby enhancing the objectivity and accuracy of outcome measures [34]. While the AI-based analysis in the included studies still relies on subjective reports and clinical symptoms [25], subsequent research could integrate such subjective data with objective physiological signals. Algorithm-driven multimodal analysis may uncover dynamic correlations between physiological data and pain characteristics, reducing biases associated with self-reports and offsetting the limitations of indirect assessment measures.

### Optimize personalized digital health interventions

Among the 18 studies included in this review, several incorporated the opinions and needs of patients with endometriosis during the development of digital health technologies for pain assessment and education. Compared with patients with other chronic conditions, individuals with endometriosis are generally younger, conferring higher accessibility and adaptability to digital health services. However, current pain intervention programs remain largely standardized and fail to fully account for interindividual differences in patients’ pain types, psychological preferences, and behavioral characteristics, thereby affecting intervention experience and adherence. First, the lack of significant effects of the activity-stimulating VR interventions on improving pain catastrophizing and negative emotions may be associated with a mismatch between activity intensity and patients’ pain tolerance [15]. All study participants were patients with moderate to severe pain, some of whom may have had limited physical mobility. The design of activity-stimulating VR failed to consider individual differences in pain tolerance, which might instead cause discomfort and reduce adherence. Second, repeated immersion in the same VR environment twice daily may lead to patient boredom and even study discontinuation [23]. Therefore, future research should scientifically design progressive intervention protocols based on pain types and objective evidence to achieve dynamic, personalized adjustments to intervention content and frequency. For example, Piette et al. [35] adopted a reinforcement learning model to dynamically adjust therapists’ interaction modes and intervention content by analyzing patients’ pain scores, activity levels, and feedback data. To improve intervention adherence, prioritizing motivation sustainability and interactive experience optimization helps strengthen patients’ self-efficacy, with real-time feedback, progress visualization, and personalized reminders as practical examples.

### Challenges and prospects of digital health technology in endometriosis pain management

While digital health technologies are advancing rapidly in pain management of patients with endometriosis, they face several challenges, which are detailed as follows: (i) Verify the long-term effectiveness: Existing studies have focused primarily on short-term outcomes, and the long-term effectiveness and sustainability of digital health technologies remain to be verified. Although *Endo* App has been shown to improve patients’ quality of life within a 12-week period [18], its long-term impacts on pain management and mechanisms underlying maintaining patient engagement remain unclear. An 8-week online mindfulness therapy intervention showed no significant effect on endometriosis-related pelvic pain, potentially due to the long disease duration of endometriosis, which makes it difficult for short-term interventions to alter pain-associated neural responses [20]. This also suggests that short-term evaluations may be insufficient to reflect the needs of long-term management for chronic conditions. Therefore, future studies should extend follow-up periods to verify the long-term effects of interventions, thereby supporting a more robust and sustainable role for digital health technologies in the management of EAP. (ii) Protect personal sensitive information: Digital health interventions typically involve collection and storage of patients’ personal information, such as symptom burden and psychological status. For individuals with endometriosis, topics such as pain and adverse sexual experiences often contribute to feelings of social isolation [36]. Without adequate safeguards, such sensitive health information is particularly vulnerable to privacy breaches, unauthorized access, and data leakage. An anonymous environment can foster a sense of safety and privacy, thereby enhancing treatment adherence. Therefore, future research should emphasize the protection of sensitive information and ensure a secure digital environment for patients. (iii) Addressing evidence gaps in adolescent populations: As frequent users of digital technologies, adolescents have unique physiological, psychological, developmental and cognitive characteristics, which offer substantial potential for digital health interventions to provide personalized care. Given the early onset of endometriosis-related symptoms and the critical importance of early pain intervention, digital health technologies are particularly promising for expanding pain education, improving symptom recognition, and supporting timely management in this population. However, current digital health tools rarely provide appropriate content matched to adolescents’ comprehension levels, and also face challenges such as risks of digital dependence, privacy concerns, and low acceptance among caregivers [37]. Future research should optimize intervention design through multidisciplinary collaboration, develop age‑appropriate pain education and psychological support content, establish strict content review and ethical safeguards, and promote coordinated implementation across schools, families, and clinical settings to prioritize the needs of adolescents. This will enable digital health technologies to provide timely, convenient, and personalized symptom management support for adolescents with endometriosis. (iv) Addressing regional bias in the evidence base and equity concerns: Current research evidence is predominantly derived from high-income countries, with insufficient data from low- and middle-income countries. Significant disparities exist between high-income countries and low- and middle-income countries in digital technology accessibility, healthcare resource allocation, and socioeconomic contexts, which restricts the global generalizability of the findings and raises important equity concerns. Future studies should expand their geographic scope, develop regionally adapted tools, and promote international collaboration to enhance the inclusivity and equity of digital health technologies for endometriosis pain management.

### Limitations

Several limitations of this scoping review should be acknowledged. First, only studies published in English and Chinese were included, which may have resulted in the omission of relevant studies in other languages. Second, despite our comprehensive search strategy, some relevant literature may not have been captured, which could affect the interpretation of our findings. Third, this study has limited the main scope of digital health technologies to patient-facing interactive tools. While this approach may not encompass the full spectrum of digital health categories (such as electronic health records that are clinician-facing only with no patient engagement), it facilitates an in-depth analysis of the shared mechanisms through which these technologies enhance patient engagement and self-management. Future reviews could expand to include a broader range of digital health technology types. Finally, as a scoping review, this study primarily aimed to systematically map and summarize the breadth of available evidence, not to formally appraise its methodological quality. It is important to note that the included studies consisted predominantly of exploratory or pilot investigations. Specifically, interventional studies were often limited by small sample sizes, single-center designs, and a lack of blinding for participants and outcome assessors. These limitations collectively highlight the evolving nature of this field and underscore the need for more robust primary studies to build a conclusive evidence base.

## Conclusion

This study systematically reviewed the research on digital health technologies for pain management in patients with endometriosis, with a focus on the content elements, application forms, and outcome indicators. The findings constitute a preliminary evidence base for the potential of these technologies in reducing pain and improving quality of life. However, challenges remain regarding the refinement of objective physiological indicators for pain assessment and the maintenance of long-term intervention effects, underscoring the early-stage nature of this research field, particularly in China. Future studies should leverage digital technologies to develop evidence-based, individualized pain management strategies and conduct long-term follow-ups to dynamically optimize intervention effects, thereby achieving sustained management of EAP.

## Supporting information

S1 Checklist Preferred Reporting Items for Systematic reviews and Meta-Analyses extension for Scoping Reviews (PRISMA-ScR) Checklist. (DOCX)

S1 Table Search strategy used for each of the databases. (DOCX)
