# Supplementary material for: Digital health technologies in pain management for patients with endometriosis: A scoping review
Source: PLoS One. 2026 Mar 25;21(3):e0345756. doi: 10.1371/journal.pone.0345756 (PMC13016302; doi:10.1371/journal.pone.0345756)
Supplement: S1 Table — (DOCX) [file pone.0345756.s002.docx]

**[T](https://journals.plos.org/plosone/article?id=10.1371/journal.pone.0322493#pone.0322493.s001)able 1. Search strategy used for each of the databases.**

| **PubMed** | |
| --- | --- |
| #1 | "endometriosis [Mesh] |
| #2 | "endometriosis"[Title/Abstract] OR "endometrioma"[Title/Abstract] OR "endometriomas"[Title/Abstract] |
| #3 | #1 OR #2 |
| #4 | "pain"[Mesh] OR "dysuria"[Mesh] OR "dyspareunia"[Mesh] |
| #5 | "pain*"[Title/Abstract] OR "dysuria"[Title/Abstract] OR "dyspareunia"[Title/Abstract] OR “dyschezia”[Title/Abstract] OR “abdominal pain”[Title/Abstract] OR "chronic pelvic pain"[Title/Abstract] |
| #6 | #4 OR #5 |
| #7 | "telemedicine"［Mesh］OR "Digital Health"[Mesh]“Internet-Based Intervention”[Mesh ] OR “Mobile Applications”[Mesh] OR “Wearable Electronic Devices”[Mesh] OR “internet of things”[Mesh] OR “Artificial Intelligence”[Mesh] OR “Virtual Reality”[Mesh] |
| #8 | “digital health technolog*”[Title/Abstract] OR “digital therapeutics”[Title/Abstract] OR “Mobile Health”[Title/Abstract] OR “mhealth”[Title/Abstract] OR “telehealth”[Title/Abstract] OR “ehealth”[Title/Abstract] OR “telecare”[Title/Abstract] OR “Web-based Intervention”[Title/Abstract] OR “Online Intervention”[Title/Abstract] OR “website”[Title/Abstract] OR “internet”[Title/Abstract] OR “Portable Software App”[Title/Abstract] OR “mobile phone”[Title/Abstract] OR “electronic handheld device”[Title/Abstract] OR “virtual”[Title/Abstract] OR “AI”[Title/Abstract] |
| #9 | #7 OR #8 |
| #10 | #3 AND #6 AND #9 |
| **Web of Science** | |
| #1 | TS=("endometriosis "OR" endometrioma "OR" endometriomas") |
| #2 | TS=("pain "OR" chronic pelvic pain "OR" dyschezia "OR" abdominal pain "OR" dysuria "OR" dysmenorrhea"OR" dyspareunia") |
| #3 | TS=("Digital Health "OR" digital health technolog* "OR" digital therapeutics "OR" Virtual Reality "OR" virtual "OR" Wearable Electronic Devices "OR" electronic handheld device "OR" Internet-Based Intervention "OR" Web-based Intervention "OR" Online Intervention "OR" telemedicine "OR" telecare "OR" telehealth "OR" Mobile Health "OR" mhealth "OR" ehealth "OR" Mobile Applications "OR" website "OR" internet "OR" mobile phone "OR" Artificial Intelligence "OR" AI "OR" Internet of Things") |
| #4 | #1 AND #2 AND #3 |
| **Cochrane** | |
| #1 | MeSH descriptor: [endometriosis] explode all trees |
| #2 | (endometrioma or endometriomas):ti,ab,kw |
| #3 | #1OR#2 |
| #4 | MeSH descriptor: [pain] explode all trees |
| #5 | MeSH descriptor: [dysuria] explode all trees |
| #6 | MeSH descriptor: [dyspareunia] explode all trees |
| #7 | (pain or dysuria or dyspareunia or dyschezia or abdominal pain or chronic pelvic pain):ti,ab,kw |
| #8 | #4OR#5OR#6OR#7 |
| #9 | #3AND#8 |
| #10 | MeSH descriptor: [Digital Health] explode all trees |
| #11 | MeSH descriptor: [Internet-Based Intervention] explode all trees |
| #12 | MeSH descriptor: [Mobile Applications] explode all trees |
| #13 | MeSH descriptor: [Telemedicine] explode all trees |
| #14 | MeSH descriptor: [Wearable Electronic Devices] explode all trees |
| #15 | MeSH descriptor: [internet of things] explode all trees |
| #16 | MeSH descriptor: [Artificial Intelligence] explode all trees |
| #17 | MeSH descriptor: [Virtual Reality] explode all trees |
| #18 | (digital health technolog or digital therapeutics or Mobile Health or mhealth or telehealth or ehealth or telecare or Web-based Intervention or Online Intervention or website or internet or Portable Software App or mobile phone or electronic handheld device or virtual or AI):ti,ab,kw |
| #19 | #10OR#11OR#12OR#13OR#14OR#15OR#16OR#17OR#18 |
| #20 | #3AND#9AND#19 |
| **Embase** | |
| #1 | 'endometriosis'/exp |
| #2 | 'endometrioma':ti,ab,kw OR 'endometriomas':ti,ab,kw |
| #3 | #1 OR #2 |
| #4 | 'pain'/exp OR 'dysuria'/exp OR dyspareunia'/exp |
| #5 | 'pain*':ti,ab,kw OR 'dysuria':ti,ab,kw OR 'dyspareunia':ti,ab,kw OR 'dyschezia':ti,ab,kw OR 'abdominal pain':ti,ab,kw OR 'chronic pelvic pain':ti,ab,kw |
| #6 | #4 OR #5 |
| #7 | 'telemedicine'/exp OR 'Digital Health'/exp 'Internet-Based Intervention'[Mesh ] OR 'Mobile Applications'/exp OR 'Wearable Electronic Devices'/exp OR 'internet of things'/exp OR 'Artificial Intelligence'/exp OR 'Virtual Reality'/exp |
| #8 | 'digital health technolog*':ti,ab,kw OR 'digital therapeutics':ti,ab,kw OR 'Mobile Health':ti,ab,kw OR 'mhealth':ti,ab,kw OR 'telehealth':ti,ab,kw OR 'ehealth':ti,ab,kw OR 'telecare':ti,ab,kw OR 'Web-based Intervention':ti,ab,kw OR 'Online Intervention':ti,ab,kw OR 'website':ti,ab,kw OR 'internet':ti,ab,kw OR 'Portable Software App':ti,ab,kw OR 'mobile phone':ti,ab,kw OR 'electronic handheld device':ti,ab,kw OR 'virtual':ti,ab,kw OR 'AI':ti,ab,kwne:ti,ab,kw |
| #9 | #7 OR #8 |
| #10 | #3 AND #6 AND #9 |
| **CINAHL** | |
| S1 | MH endometriosis |
| S2 | TI ( " endometrioma "OR" endometriomas" ) |
| S3 | S1 OR S2 |
| S4 | MH ("pain "OR" dysuria "OR" dyspareunia") |
| S5 | TI ("pain "OR" chronic pelvic pain "OR" dyschezia "OR" abdominal pain "OR" dysuria "OR" dysmenorrhea"OR" dyspareunia") |
| S6 | S4 OR S5 |
| S7 | MH ("telemedicine" OR "Digital Health""Internet-Based Intervention" OR "Mobile Applications” OR "Wearable Electronic Devices” OR "internet of things” OR "Artificial Intelligence” OR "Virtual Reality”) |
| S8 | TI ("digital health technolog*" OR "digital therapeutics" OR "Mobile Health" OR "mhealth" OR "telehealth" OR "ehealth" OR "telecare" OR "Web-based Intervention" OR "Online Intervention" OR "website" OR "internet" OR "Portable Software App" OR "mobile phone" OR "electronic handheld device" OR "virtual" OR "AI" ) |
| S9 | S7 OR S8 |
| S10 | S3 AND S6 AND S9 |
| **China National Knowledge Infrastructure (CNKI) (Chinese)** | |
| （Subject:"endometriosis") and （Subject: "pain" or "dysmenorrhea" or "chronic pelvic pain" or "dyspareunia" or "acute abdominal pain" or "dyschezia" or "dysuria"）and （Subject: "mobile health" or "telehealth" or "digital health" or "e-health" or "web-based intervention" or "internet" or "computer" or "website" or "mobile platform" or "software" or "online" or "mobile application" or "WeChat" or "wearable device" or "virtual reality" or "artificial intelligence" or "big data" or "internet of things"） | |
| **WANFANG DATA (Chinese)** | |
| （Subject:"endometriosis") and （Subject: "pain" or "dysmenorrhea" or "chronic pelvic pain" or "dyspareunia" or "acute abdominal pain" or "dyschezia" or "dysuria"）and （Subject: "mobile health" or "telehealth" or "digital health" or "e-health" or "web-based intervention" or "internet" or "computer" or "website" or "mobile platform" or "software" or "online" or "mobile application" or "WeChat" or "wearable device" or "virtual reality" or "artificial intelligence" or "big data" or "internet of things"） | |
| **China Biomedical Literature Database (Chinese)** | |
| （Subject:"endometriosis") and （Subject: "pain" or "dysmenorrhea" or "chronic pelvic pain" or "dyspareunia" or "acute abdominal pain" or "dyschezia" or "dysuria"）and （Subject: "mobile health" or "telehealth" or "digital health" or "e-health" or "web-based intervention" or "internet" or "computer" or "website" or "mobile platform" or "software" or "online" or "mobile application" or "WeChat" or "wearable device" or "virtual reality" or "artificial intelligence" or "big data" or "internet of things"） | |
